# Association between traditional fermented milk (*mursik*) consumption and oesophageal carcinoma among patients presenting to two referral hospitals in Nandi County, Kenya: A case-control study

**DOI:** 10.1371/journal.pone.0324412

**Published:** 2025-09-29

**Authors:** Rancy Chepkoech Mutai, Marshal M. Mweu

**Affiliations:** Department of Public and Global Health, Faculty of Health Sciences, University of Nairobi, Nairobi, Kenya; University of the Punjab Quaid-i-Azam Campus: University of the Punjab, PAKISTAN

## Abstract

**Background:**

Oesophageal carcinoma (OC) is a prominent cause of morbidity and mortality in low and middle-income countries yet little evidence exists about its contextual drivers. The objectives of this study were to assess the association between traditional fermented milk (FM) (*mursik*) consumption and OC independent of known socio-demographic and lifestyle risk factors as well as to quantify its population impact among patients presenting to two referral hospitals in Nandi County, Kenya.

**Methods:**

A hospital-based case-control study was employed to assess the FM-OC relationship among patients presenting to Kapsabet Surgical Care Centre and Kapsabet County Referral Hospital in Nandi County, Kenya for care between 23^rd^ November 2023 and 13^th^ January 2024. All 33 cases meeting specific eligibility criteria were prospectively recruited whilst 131 controls were simple randomly sampled and frequency-matched to the cases by hospital and day of presentation. A logistic regression model was fitted to assess the FM-OC association while adjusting for potential confounders. Subsequently, a population attributable fraction (PAF) for the association (along with its confidence interval) was estimated.

**Results:**

A strong association between FM and OC was noted; the odds of OC among frequent and infrequent consumers of FM being over nine (OR 9.1, 95% CI: 3.1–26.6) and three (OR 3.2, 95% CI: 1.1–9.2) times higher, respectively, than non-consumers. This association was not substantially confounded by the studied socio-demographic and lifestyle factors. The PAF estimate for this association was 65.2% (95% CI: 40.1–90.7).

**Conclusions:**

In this study setting, *mursik* consumption was strongly associated with OC independent of other risk factors. This association registered a high PAF suggesting that up to 65% of OC in the population could be prevented if *mursik* was not consumed. This finding calls for safe, community-owned alternatives for fermenting milk in order to mitigate the risk of OC in this population.

## Introduction

Gastrointestinal (GI) cancers constitute a major public health concern accounting for 26% of the global cancer incidence and 35% of all cancer-related deaths in 2018 [1]. Of GI cancers, oesophageal carcinoma (OC) features prominently particularly in low and middle-income countries (LMICs) [2]. OC is a malignancy of the oesophagus typically characterised by dysphagia, odynophagia, unexplained weight loss, chest pain, worsening dyspepsia, and chronic cough [3].

Globally, age-standardised incidence rates of OC are highest in Asia and Africa – standing at 6.2 and 3.6, per 100,000 respectively, as of 2022 [4]. In Sub-Saharan Africa, the rates are highest in the ‘oesophageal cancer corridor’ [5,6], which comprises Ethiopia (4.97/10^5^), Kenya (11.75/10^5^), Uganda (14.81/10^5^), Tanzania (11.36/10^5^), Malawi (26.06/10^5^) and South Africa (10.43/10^5^) [7]. Within Kenya, the highest burden is registered in the Rift Valley region in the western part of the country [8]; the 5-year survival rate being 25% [9].

Fermented milk (FM) (commonly known as *mursik*) remains a favourite cultural beverage among the indigenous inhabitants of Kenya’s Rift Valley [10,11]. It is prepared by boiling and fermenting fresh cow’s or goat’s milk for a period of 3–5 days, and thereafter, charcoal powder is added [10,11]. Despite the health benefits of fermented milk, its consumption has been linked to the occurrence of OC [12] ascribable to improper milk handling and fermentation processes which may introduce contaminants such as moulds that produce various mycotoxins [10,13]. These toxins may contribute to oesophageal carcinogenesis when ingested habitually [11,13]. Moreover, charcoal as a key ingredient is believed to harbour polycyclic aromatic hydrocarbons (PAHs) [14] that could catalyse the carcinogenicity of *mursik*.

Besides FM, alcohol and tobacco use, biomass fuels, socio-demographic characteristics and hot beverage consumption are well-recognised risk factors for OC [8,12,15–19]. Alcoholic drinks may contain acetaldehydes and nitrosamines that are carcinogenic [12,20]. PAHs, aldehydes, and nitrosamines released during tobacco smoking may precipitate carcinogenesis [21]. Wood fuel, ubiquitously used in Kenyan rural households, is known to emit PAHs, benzene, arsenic, 1,3-butadiene and formaldehyde that can trigger oesophageal carcinogenesis [18,19,22,23]. The risk of OC increases with age, with individuals aged ≥50 years bearing a disproportionate risk [8]. OC is more common in males than females – attributable to higher rates of tobacco smoking and alcohol use among males [24,25]. Consumption of hot beverages >65^0^c could potentially be carcinogenic [26]. Hot drinks may instigate thermal injury of the oesophageal mucosa – a risk for OC [27,28].

Despite OC’s preponderance in LMICs, there is scarcity of evidence on its contextual drivers in endemic settings. Consequently, the objectives of this study were to quantify the association between FM consumption and OC independent of known risk factors as well as to estimate FM’s population impact among patients presenting to two referral hospitals in Nandi County, Kenya. An understanding of FM’s role in the occurrence of OC is critical to guiding policy development and informing the design of appropriate interventions for the control of OC in Kenya.

## Materials and methods

### Study setting, design and population

The study was carried out at Kapsabet Surgical Care Centre (KSCC) and Kapsabet County Referral Hospital (KCRH) situated in Kapsabet town, Nandi County within the Rift Valley region of Kenya. KSCC is a private hospital specialising in endoscopy and surgical care whereas KCRH is a state-run referral facility offering a range of preventive and curative services. Notably, the two hospitals serve a catchment population of ~ 885,700 persons comprising majorly of Nandi natives of the Kalenjin community [29]. Of note, subsistence and commercial crop (tea and maize) and dairy farming are the economic mainstay of this region.

A hospital-based case-control study design was employed in this study to evaluate the association between FM consumption and OC. The rationale for the choice of the design stems not only from its suitability for studying rare outcomes but also the ease and efficiency of recruitment of cases presenting to a hospital setting for care. Importantly, the study conformed to STROBE guidelines for reporting of case-control studies [30].

The study population comprised County-resident patients aged ≥18 years presenting to KSCC and KCRH for care between 23^rd^ November 2023 and 13^th^ January 2024 and who had consented to participate in the study. Critically-ill patients and those uninterviewable and/or unable to give consent were excluded from the study.

### Outcome definition

Cases were eligible individuals attending the facilities with any suggestive symptoms: dysphagia, odynophagia, worsening dyspepsia, persistent coughing, chest pains and unexplained weight loss and had a confirmed diagnosis of OC by endoscopic biopsy and histopathological examination during the study period. Controls were patients presenting to the same facilities as cases but with other conditions not thought to be related to *mursik* consumption. As such, those with gastrointestinal disorders were excluded with a view to minimise selection bias [31].

### Sample size and participant selection

The size of the study sample was estimated as suggested by Kelsey *et al*. [32] for case control studies:


$$n_{\text{1}}=\frac{\left(Z_{\alpha }+\;Z_{\beta }{)}^{\text{2}}\overset{―}{p}\;\overset{―}{q}(r+\text{1}\right)}{r(p_{\text{1}}-p_{\text{2}}{)}^{\text{2}}}$$

$$p_{\text{1}}=\frac{p_{\text{2}}OR}{\text{1}+p_{\text{2}}(OR-\text{1})}$$

$$n_{\text{2}}=rn_{\text{1}}$$

$$\overset{―}{p}=\frac{p_{\text{1}}+rp_{\text{2}}}{r+\text{1}}$$

$$\;and\;\overset{―}{q}=\text{1}-\overset{―}{p}$$

Where $Z_{\alpha }$ = 1.96 is the required value for a two-tailed 95% confidence level; $Z_{\beta }\;$ = −0.84 is the desired value for an 80% statistical power; $n_{\text{1}}$ is the number of cases; $n_{\text{2}}$ is the number of controls; *r* = 4 is the ratio of controls to cases; $p_{\text{1}}$ is the proportion of cases consuming FM; $p_{\text{2}}$ is the proportion of controls that consume FM – specified at 10.5% based on a previous study [12] and OR is the odds ratio for the FM-OC association (set at 3.72) informed by literature [12]. Given an anticipated non-response rate of 5%, the necessary sample was 164 subjects: 33 cases and 131 controls. All eligible cases were prospectively recruited over the study period within the facilities until the desired number was attained. Controls were simple randomly sampled from a sampling frame of patients admitted at the facilities’ medical and surgical wards and frequency-matched to cases by hospital and day of presentation.

### Study variables

The primary exposure in this study was the respondent’s typical FM consumption habits. The other exposures considered included the participants’ sociodemographic characteristics (age, sex, education level, employment status and marital status), lifestyle factors (alcohol, tobacco use and beverage temperature) and cooking fuel. These variables were measured using an orally administered semi-structured questionnaire captured in both English and Swahili. Notably, interviews were conducted immediately following recruitment of study participants. Table 1 displays the assessment of these variables.

**Table 1:** Measurement of the study variables.

Variable (type)	Measurement
Fermented milk (ordinal)	The respondent’s usual consumption frequency of fermented milk over one year (prior to the interview) was captured in three categories: non-consumer, infrequent (1–2 times a week) and frequent (≥ 3 times a week)
Alcohol (ordinal)	The respondent’s usual consumption frequency of alcohol over one year was classified into three categories: non-consumer, infrequent (1–2 times a week) and frequent (≥ 3 times a week)
Cooking fuel (nominal)	This reflected the respondent’s primary type of cooking fuel. Categorised into three: biomass fuels, natural/biogas and kerosene
Tobacco (nominal)	Assessed on the basis of smoking or chewing of tobacco over one year: user or non-user.
Beverage temperature (ordinal)	This was premised on the respondent’s preferred consumption temperature of beverages. Classified into three categories: warm, hot and very hot.
Age (continuous)	Captured in years.
Sex (nominal)	Captured as male or female.
Marital status (nominal)	Assessed in four categories: single, married, divorced/separated or widowed
Employment status (nominal)	Captured in two categories: employed or unemployed.
Education level (ordinal)	This reflected an individual’s highest level of education: no formal education, primary, secondary and tertiary levels.

### Ethical considerations

Written permission to conduct the study was secured from the Kenyatta National Hospital-University of Nairobi Ethics and Research Committee (PF26/06/2023), the National Commission for Science, Technology and Innovation (NACOSTI/P/23/30502) and the Nandi County Health Services (Ref. No. CDH/NDI/2021/R.A/5). Furthermore, written informed consent was obtained from the participants prior to enlisting them in the study. In addition, the data collected were analyzed anonymously.

### Minimisation of biases

Before data collection, two research assistants were trained on standardised interviewing techniques in order to limit interviewer bias during elicitation of information from respondents. Since differential recall of consumption habits (FM, alcohol and beverage) between cases and controls was likely, questioning on these related to recent consumption. Moreover, as the likelihood of altering consumption patterns by cases was probable, reverse causality was minimised by focusing on incident cases, with questioning on typical consumption habits directed at the period prior to the onset of clinical symptoms.

### Statistical analysis

Upon data collection, the questionnaire responses were examined for completeness, after which qualitative variables were coded. Subsequently, the data were entered into a Microsoft Excel spreadsheet and cross-checked for accuracy. The dataset was then exported to R software v4.4.0 for cleaning and analysis. The R code for these analyses is available as supporting information [33]. For descriptive statistics, medians and ranges were computed for continuous variables whereas frequencies and percentages summarised categorical variables. A logistic regression model was fitted to evaluate the crude association between FM and OC. To evaluate the study covariates (age, sex, education level, employment status, marital status, alcohol, cooking fuel, tobacco and beverage temperature) as potential confounders of the FM–OC association, each of these factors was separately screened for its association with OC and FM using the Fisher’s exact test at *P*<0.05 (this two-stage testing process is synonymous with sequential screening where only variables associated with the outcome at *P*<0.05 are subjected to subsequent assessment with the primary exposure). For analytical ease, age was grouped into three categories: ≤ 40 years, 41–65 years, > 65 years and cooking fuel was reclassified into two categories: biomass/kerosene and natural/biogas since there were no cases using kerosene. Qualifying covariates from this sequential screening were deemed potential confounders of the FM–OC relationship and thus included in a multivariable logistic regression model to adjust for their confounding effect. At this stage, only those variables that resulted in a >20% change in the coefficient for FM following a backward step-wise elimination procedure were considered important confounders and thus retained in the final model [31].

A population attributable fraction (PAF) – a measure quantifying the proportion of disease in the population that is preventable if the exposure were eliminated [31] – was computed from estimates of the final model [34]:


$$PAF=\sum_{i}{pd}_{i}\left(\frac{{aOR}_{i}-\text{1}}{{aOR}_{i}}\right)$$

Where: ${pd}_{i}$ reflects the proportion of cases consuming FM infrequently and frequently (non-baseline categories) and ${aOR}_{i}$ is the specific adjusted odds ratio for the non-baseline levels of FM. Estimation of PAF (along with its bootstrapped confidence interval) was realised using the *graphPAF* package [35] written in R software.

## Results

Of the sample of 164 subjects, 160 (32 cases and 128 controls) gave consent to the study. Descriptive statistics for the study exposure variables are indicated in Table 2. The respondents’ median age was 60 years (Range: 30–78) for cases and 50 years (Range: 21–98) for controls. On FM, 46.9% (*n* = 15) of cases were frequent consumers compared with 14.8% (*n* = 19) of controls. About 28.1% (*n* = 9) of cases were frequent consumers of alcohol in comparison with 10.2% (*n* = 13) of controls.

**Table 2:** Summary statistics for the socio-demographic and lifestyle characteristics of the respondents.

Variable	Category	Cases: *n* (%)	Controls: *n* (%)
Age in years	Median (Range)*	60 (30–78)	50 (21–98)
Sex	Male	21 (65.6)	60 (46.9)
	Female	11 (34.4)	68 (53.1)
Marital status	Single	2 (6.3)	29 (22.7)
	Married	24 (75.0)	85 (66.4)
	Divorced/Separated	4 (12.5)	6 (4.7)
	Widowed	2 (6.3)	8 (6.3)
Education level	No formal education	4 (12.5)	11 (8.6)
	Primary	21 (65.6)	48 (37.5)
	Secondary	6 (18.8)	35 (27.3)
	Tertiary	1 (3.1)	34 (26.6)
Employment status	Unemployed	15 (46.9)	56 (43.8)
	Employed	17 (53.1)	72 (56.3)
Alcohol	Non-consumer	10 (31.2)	85 (66.4)
	Infrequent	13 (40.6)	30 (23.4)
	Regular	9 (28.1)	13 (10.2)
Tobacco	Non-user	17 (53.1)	109 (85.2)
	User	15 (46.9)	19 (14.8)
Beverage temperature	Warm	18 (56.2)	66 (51.6)
	Hot	7 (21.9)	41 (32.0)
	Very hot	7 (21.9)	21 (16.4)
Fermented milk	Non-consumer	6 (18.8)	69 (53.9)
	Infrequent	11 (34.4)	40 (31.3)
	Frequent	15 (46.9)	19 (14.8)
Cooking fuel	Biomass	27 (84.4)	83 (64.8)
	Kerosene	0 (0.0)	8 (6.3)
	Natural/biogas	5 (15.6)	37 (28.9)

*Median (Range): only applies to age

The crude association between FM and OC is captured in Table 3. The odds of OC were approximately three (3.2; 95% CI: [1.1–9.2]) and nine (9.1; 95% CI: [3.1–26.6]) times higher amongst infrequent and frequent consumers, respectively, than non-consumers.

**Table 3:** Univariable analysis of the association between fermented milk consumption and oesophageal carcinoma among patients at KSCC and KCRH, Nandi County, Kenya.

Variable	Category	cOR*	95% CI	*P*-value
Fermented milk	Non-consumer	Ref	–	<0.001
	Infrequent	3.2	1.1; 9.2	
	Frequent	9.1	3.1; 26.6	

*Crude odds ratio

The association between individual covariates and OC is presented in Table 4. Notably, age group, education level, alcohol and tobacco were associated with OC at the 5% significance level. As such, these variables qualified for assessment of their association with FM.

**Table 4:** Association between individual covariates and oesophageal carcinoma among patients attending KSCC and KCRH, Nandi County, Kenya.

Variable	Category	Cases: *n* (%)	Controls: *n* (%)	*P*-value
*Age group	≤ 40 years	2 (6.3)	42 (32.8)	0.005
	41-65 years	21 (65.6)	57 (44.5)	
	>65 years	9 (28.1)	29 (22.7)	
Sex	Male	21 (65.6)	60 (46.9)	0.075
	Female	11 (34.4)	68 (53.1)	
*Education level	No formal education	4 (12.5)	11 (8.6)	0.003
	Primary	21 (65.6)	48 (37.5)	
	Secondary	6 (18.8)	35 (27.3)	
	Tertiary	1 (3.1)	34 (26.6)	
Employment status	Unemployed	15 (46.9)	56 (43.8)	0.843
	Employed	17 (53.1)	72 (56.2)	
Marital status	Single	2 (6.3)	29 (22.7)	0.066
	Married	24 (75.0)	85 (66.4)	
	Divorced/separated	4 (12.5)	6 (4.7)	
	Widowed	2 (6.3)	8 (6.3)	
*Alcohol	Non-consumers	10 (31.3)	85 (66.4)	0.001
	Infrequent	13 (40.6)	30 (23.4)	
	Frequent	9 (28.1)	13 (10.2)	
Cooking fuel	Biomass/kerosene	27 (84.4)	91 (71.1)	0.177
	Natural/biogas	5 (15.6)	37 (28.9)	
*Tobacco	Non-users	17 (53.1)	109 (85.2)	<0.001
	users	15 (46.9)	19 (14.8)	
Beverage temperature	Warm	18 (56.3)	66 (51.6)	0.480
	Hot	7 (21.9)	41 (32.0)	
	Very hot	7 (21.9)	21 (16.4)	

*Eligible for screening for their association with fermented milk (*p* < 0.05)

Table 5 presents the results for the association between qualifying covariates and FM. Importantly, only age group and alcohol were significantly associated with fermented milk (*p* < 0.05). Consequently, these variables were deemed potential confounders of the FM-OC association and were offered to a multivariable logistic regression model to adjust for their confounding effect.

**Table 5:** Association between qualifying exposures and fermented milk consumption among patients attending KSCC and KCRH, Nandi County, Kenya.

Variable	Category	Non-consumers: *n* (%)	Infrequent consumers: *n* (%)	Frequent consumers: *n* (%)	*P*-value
*Age group	≤ 40 years	26 (34.7)	12 (23.5)	6 (17.6)	0.038
	41-65 years	30 (40.0)	32 (62.7)	16 (47.1)	
	>65 years	19 (25.3)	7 (13.7)	12 (35.3)	
Education level	No formal education	6 (8.0)	4 (7.8)	5 (14.7)	0.096
	Primary	27 (36.0)	21 (41.2)	21 (61.8)	
	Secondary	22 (29.3)	14 (27.5)	5 (14.7)	
	Tertiary	20 (26.7)	12 (23.5)	3 (8.8)	
*Alcohol	Non-consumers	54 (72.0)	31 (60.8)	10 (29.4)	0.001
	Infrequent	15 (20.0)	13 (25.5)	15 (44.1)	
	Frequent	6 (8.0)	7 (13.7)	9 (26.5)	
Tobacco	Non-users	63 (84.0)	39 (76.5)	24 (70.6)	0.250
	users	12 (16.0)	12 (23.5)	10 (29.4)	

*Eligible for inclusion in the multivariable logistic regression model (*p* < 0.05)

Results of the multivariable analysis of the FM-OC association are presented in Table 6. Since alcohol and age group did not confound the FM-OC association (i.e., neither of the factors resulted in a >20% change in the coefficient for FM) the odds ratio for the unadjusted association was maintained and subsequently employed in the derivation of PAF. The PAF estimate for the FM-OC association was 65.2% (95% CI: 40.1–90.7).

**Table 6:** Multivariable analysis of the association between fermented milk and oesophageal carcinoma among patients at KSCC and KCRH, Nandi County, Kenya.

Variable	Category	aOR*	95% CI	*P*-value
Fermented milk	Non-consumer	Ref	–	0.005
	Infrequent	2.5	0.8; 7.7	
	Frequent	6.1	2.0; 19.2	
Alcohol	Non-consumer	Ref	–	0.085
	Infrequent	2.4	0.9; 6.6	
	Frequent	3.3	1.0; 10.8	
Age group	≤ 40 years	Ref	–	0.026
	41-65 years	6.3	1.3; 30.3	
	>65 years	4.2	0.8; 22.9	

*Adjusted odds ratio

## Discussion

In this population, an analysis of the association between FM consumption and OC has demonstrated a robust relationship. The odds of OC were roughly three and nine times higher amongst infrequent and frequent consumers, respectively, than non-consumers; signifying a dose-response relationship. In particular, this association was not confounded by any of the examined covariates. Patel *et al.* [12] corroborate this strong relationship – observing about four times higher odds of OC amongst *mursik* consumers. Importantly, markedly high concentrations of ethanol (>100mM) and acetaldehyde (>1800µM) have been detected previously in *mursik* [13] – carcinogenicity of acetaldehyde being exhibited in both animal and in vitro models at fairly low concentrations (100 µM) [36,37] Conceivably, recurrent exposure of the oesophageal mucosa to these mutagenic substances in *mursik* may explain its carcinogenicity. In Afghanistan, a linkage between consumption of fermented dairy products (cheese and yoghurt) and OC has been established [38].

As sterility during the traditional fermentation process is not guaranteed, contamination of *mursik* by fungal species of the genera *Aspergillus*, *Penicillium* and *Fusarium* is likely, with resultant production of mycotoxins [10]. Of these, aflatoxin is the predominant metabolite recovered from *mursik* [39]*.* Notably, aflatoxins have been implicated in oesophageal carcinogenesis [40].

Customarily, fermentation gourds for *mursik* are lined with specially ground fine charcoal that imparts a smoky flavour [41]. Nonetheless, charcoal could be a source of PAHs – potentially carcinogenic – spurring oesophageal carcinogenesis [14]. More so, charcoal-derived carcinogens have been pointed out as contributory to the high incidence of OC in Iran [42].

Despite our finding of a significant elevated risk of OC with FM consumption, some studies have posted contradictory findings [21,43]. In particular, Oncina-Cánovas *et al.* [43] report a protective association between consumption of fermented dairy products and OC. This divergence of observations across study contexts underscores the need to carefully examine the role played by disparate dairy fermentation processes in driving local and/or regional OC burdens.

The PAF estimate of 65.2% for FM in this study implies that roughly two-thirds of OC in the study population could potentially be prevented if FM was not consumed. Irrefutably, FM as a singular factor is impactful in this population – contributing significantly to the OC burden in this setting. This highlights the necessity of raising awareness on general safety practices for preparing fermented milk.

A few limitations are inherent in this study. Despite efforts to adjust the association for known confounders, the possibility of residual confounding persists. Other unmeasurable factors such as genetic predispositions and environmental exposures could still have influenced the observed relationship between FM and OC. For instance, polymorphisms of ethanol metabolising enzymes (alcohol dehydrogenase and aldehyde dehydrogenase) have shown significant relation to alcohol intake [44] and may contribute to the occurrence of OC [13]. Although the recruitment of controls from hospital settings not only affords convenience but also controls for potential differences in health-seeking behaviours between cases and controls, their utility may introduce selection bias. Hospitalised controls generally tend to have higher exposures compared to the general population that they represent. Consequently, for our study, this undertaking is likely to have weakened the FM-OC association.

## Conclusions

This study has revealed a strong association between *mursik* consumption and OC in this setting. This relationship was not confounded by any of the studied factors. The association yielded a high PAF implying that up to 65% of OC in the population could be prevented if *mursik* was not consumed. This finding calls for dedicated campaigns to raise local awareness with a view to develop safe, community-acceptable fermentation processes to mitigate OC risk.

## References

[1] Arnold M, Abnet CC, Neale RE, Vignat J, Giovannucci EL, McGlynn KA, et al. Global Burden of 5 Major Types of Gastrointestinal Cancer. Gastroenterology. 2020;159(1):335–349.e15. doi: 10.1053/j.gastro.2020.02.068 32247694 PMC8630546

[2] GBD 2017 Oesophageal Cancer Collaborators. The global, regional, and national burden of oesophageal cancer and its attributable risk factors in 195 countries and territories, 1990-2017: a systematic analysis for the Global Burden of Disease Study 2017. Lancet Gastroenterol Hepatol. 2020;5(6):582–97. doi: 10.1016/S2468-1253(20)30007-8 32246941 PMC7232026

[3] Ellis A, Risk JM, Maruthappu T, Kelsell DP. Tylosis with oesophageal cancer: Diagnosis, management and molecular mechanisms. Orphanet J Rare Dis. 2015;10:126. doi: 10.1186/s13023-015-0346-2 26419362 PMC4589029

[4] Teng Y, Xia C, Cao M, Yang F, Yan X, He S, et al. Esophageal cancer global burden profiles, trends, and contributors. Cancer Biol Med. 2024;21. doi: 10.20892/j.issn.2095-3941.2024.0145 PMC11359494 39066471

[5] Schaafsma T, Wakefield J, Hanisch R, Bray F, Schüz J, Joy EJM, et al. Africa’s oesophageal cancer corridor: geographic variations in incidence correlate with certain micronutrient deficiencies. PLoS One. 2015;10(10):e0140107. doi: 10.1371/journal.pone.0140107 26448405 PMC4598094

[6] Kayamba V. Oesophageal cancer hotspots in Africa. Lancet Gastroenterol Hepatol. 2019;4(11):818–20. doi: 10.1016/S2468-1253(19)30253-5 31609235

[7] Zhang C, Chen L, Xiu Y, Zhang H, Zhang Y, Ying W. Burden of esophageal cancer in global, regional and national regions from 1990 to 2021 and its projection until 2050: results from the GBD study 2021. Front Oncol. 2025;14:1518567. doi: 10.3389/fonc.2024.1518567 39902130 PMC11788179

[8] Odera JO, Odera E, Githang’a J, Walong EO, Li F, Xiong Z, et al. Esophageal cancer in Kenya. Am J Dig Dis (Madison). 2017;4(3):23–33. 29082268 PMC5659304

[9] Degu A, Karimi PN, Opanga SA, Nyamu DG. Determinants of survival outcomes among esophageal cancer patients at a national referral hospital in Kenya. Chronic Dis Transl Med. 2022;9(1):20–8. doi: 10.1002/cdt3.52 36926251 PMC10011667

[10] John MN, Joseph WM, Zacchaeus ON, Moses BS. Spontaneously fermented kenyan milk products: a review of the current state and future perspectives. Afr J Food Sci. 2017;11(1):1–11. doi: 10.5897/ajfs2016.1516

[11] Kigen G, Busakhala N, Kamuren Z, Rono H, Kimalat W, Njiru E. Factors associated with the high prevalence of oesophageal cancer in Western Kenya: a review. Infect Agent Cancer. 2017;12:59. doi: 10.1186/s13027-017-0169-y 29142587 PMC5670732

[12] Patel K, Wakhisi J, Mining S, Mwangi A, Patel R. Esophageal cancer, the topmost cancer at MTRH in the Rift Valley, Kenya, and Its Potential Risk Factors. ISRN Oncol. 2013;2013:1–9. doi: 10.1155/2013/503249 PMC3893746 24490085

[13] Nieminen MT, Novak-Frazer L, Collins R, Dawsey SP, Dawsey SM, Abnet CC, et al. Alcohol and acetaldehyde in African fermented milk mursik--a possible etiologic factor for high incidence of esophageal cancer in western Kenya. Cancer Epidemiol Biomarkers Prev. 2013;22(1):69–75. doi: 10.1158/1055-9965.EPI-12-0908 23155139 PMC3538938

[14] Liu G, Niu Z, Van Niekerk D, Xue J, Zheng L. Polycyclic aromatic hydrocarbons (PAHs) from coal combustion: emissions, analysis, and toxicology. Rev Environ Contam Toxicol. 2008;192:1–28. doi: 10.1007/978-0-387-71724-1_1 18020302

[15] Yang J, Liu X, Cao S, Dong X, Rao S, Cai K. Understanding esophageal cancer: the challenges and opportunities for the next decade. Front Oncol. 2020;10:1727. doi: 10.3389/fonc.2020.01727 33014854 PMC7511760

[16] Domper Arnal MJ, Ferrández Arenas Á, Lanas Arbeloa Á. Esophageal cancer: risk factors, screening and endoscopic treatment in Western and Eastern countries. World J Gastroenterol. 2015;21(26):7933–43. doi: 10.3748/wjg.v21.i26.7933 26185366 PMC4499337

[17] Saadaat R, Abdul-Ghafar J, Hanifi AN, Khalid S, Khairy AL, Ibrahimkhil AS, et al. Risk factors associated with esophageal cancers, diagnosed at tertiary level in Afghanistan: a descriptive cross-sectional study. BMC Cancer. 2022;22(1):1112. doi: 10.1186/s12885-022-10228-9 36316690 PMC9623968

[18] Okello S, Akello SJ, Dwomoh E, Byaruhanga E, Opio CK, Zhang R, et al. Biomass fuel as a risk factor for esophageal squamous cell carcinoma: a systematic review and meta-analysis. Environ Health. 2019;18(1):60. doi: 10.1186/s12940-019-0496-0 31262333 PMC6604279

[19] Josyula S, Lin J, Xue X, Rothman N, Lan Q, Rohan TE, et al. Household air pollution and cancers other than lung: a meta-analysis. Environ Health. 2015;14:24. doi: 10.1186/s12940-015-0001-3 25890249 PMC4377187

[20] Yang CS, Chen XL. Research on esophageal cancer: With personal perspectives from studies in China and Kenya. Int J Cancer. 2021;149(2):264–76. doi: 10.1002/ijc.33421 33270917 PMC8141013

[21] Wang T, Zhu Y, Zheng Y, Cao Y, Xu Q, Wang X, et al. Dairy consumption and risk of esophagus cancer in the prostate, lung, colorectal, and ovarian cohort. Front Nutr. 2022;9. doi: 10.3389/fnut.2022.1015062 PMC9773090 36570164

[22] Gauggel-Lewandowski S, Heussner AH, Steinberg P, Pieterse B, van der Burg B, Dietrich DR. Bioavailability and potential carcinogenicity of polycyclic aromatic hydrocarbons from wood combustion particulate matter in vitro. Chem Biol Interact. 2013;206(2):411–22. doi: 10.1016/j.cbi.2013.05.015 23796820

[23] Gustafson P, Barregard L, Strandberg B, Sällsten G. The impact of domestic wood burning on personal, indoor and outdoor levels of 1,3-butadiene, benzene, formaldehyde and acetaldehyde. J Environ Monit. 2007;9(1):23–32. doi: 10.1039/b614142k 17213939

[24] Nasrollahzadeh D, Kamangar F, Aghcheli K, Sotoudeh M, Islami F, Abnet CC, et al. Opium, tobacco, and alcohol use in relation to oesophageal squamous cell carcinoma in a high-risk area of Iran. Br J Cancer. 2008;98(11):1857–63. doi: 10.1038/sj.bjc.6604369 18475303 PMC2410115

[25] Liu C-Q, Ma Y-L, Qin Q, Wang P-H, Luo Y, Xu P-F, et al. Epidemiology of esophageal cancer in 2020 and projections to 2030 and 2040. Thorac Cancer. 2023;14(1):3–11. doi: 10.1111/1759-7714.14745 36482832 PMC9807450

[26] Loomis D, Guha N, Hall AL, Straif K. Identifying occupational carcinogens: an update from the IARC Monographs. Occup Environ Med. 2018;75(8):593–603. doi: 10.1136/oemed-2017-104944 29769352 PMC6204931

[27] Luo H, Ge H. Hot tea consumption and esophageal cancer risk: a meta-analysis of observational studies. Front Nutr. 2022;9:831567. doi: 10.3389/fnut.2022.831567 35479756 PMC9035825

[28] Chen Y, Tong Y, Yang C, Gan Y, Sun H, Bi H, et al. Consumption of hot beverages and foods and the risk of esophageal cancer: a meta-analysis of observational studies. BMC Cancer. 2015;15:449. doi: 10.1186/s12885-015-1185-1 26031666 PMC4457273

[29] Kenya National Bureau of Statistics. Kenya population and housing census volume 1: Population by County and sub-County. Kenya National Bureau of Statistics. 2019. Available from: https://www.knbs.or.ke/?wpdmpro=2019-kenya-population-and-housing-census-volume-i-population-by-county-and-sub-county

[30] von Elm E, Altman DG, Egger M, Pocock SJ, Gøtzsche PC, Vandenbroucke JP, et al. The Strengthening the Reporting of Observational Studies in Epidemiology (STROBE) statement: guidelines for reporting observational studies. J Clin Epidemiol. 2008;61(4):344–9. doi: 10.1016/j.jclinepi.2007.11.008 18313558

[31] Dohoo IR, Martin W, Stryhn H. Methods in Epidemiologic Research. 1st ed. Charlottetown, P.E.I.: VER, Inc.; 2012.

[32] Jennifer KL, Alice WS, Alfred ES, Douglas TW. Methods in Observational Epidemiology. 2nd Ed. New York: Oxford University Press, Inc.; 1996. https://books.google.co.in/books?id=Xnz6VgL22osC&printsec=frontcover&source=gbsgesummaryr&cad=0#v=onepage&q&f=false

[33] MutaiR. Replication Data: Association between fermented milk consumption and oesophageal carcinoma among patients presenting to two referral hospitals in Nandi County, Kenya: a case control study. DRAFT VERSION ed: Harvard Dataverse. 2025. pp. 1. doi: 10.7910/DVN/DC0IMK41021552

[34] Rockhill B, Newman B, Weinberg C. Use and misuse of population attributable fractions. Am J Public Health. 1998;88(1):15–9. doi: 10.2105/ajph.88.1.15 9584027 PMC1508384

[35] Ferguson J, O’Connell M. Estimating and displaying population attributable fractions using the R package: graphPAF. Eur J Epidemiol. 2024;39(7):715–42. doi: 10.1007/s10654-024-01129-1 38971917 PMC11343908

[36] Lachenmeier DW, Sohnius E-M. The role of acetaldehyde outside ethanol metabolism in the carcinogenicity of alcoholic beverages: evidence from a large chemical survey. Food Chem Toxicol. 2008;46(8):2903–11. doi: 10.1016/j.fct.2008.05.034 18577414

[37] Seitz HK, Stickel F. Molecular mechanisms of alcohol-mediated carcinogenesis. Nat Rev Cancer. 2007;7(8):599–612. doi: 10.1038/nrc2191 17646865

[38] Eser S, Özgür S, Shayan NA, Abdianwall MH. Risk Factors Related to Esophageal Cancer, a Case-Control Study in Herat Province of Afghanistan. Arch Iran Med. 2022;25(10):682–90. doi: 10.34172/aim.2022.107 37542400 PMC10685874

[39] Talaam KK, Nga’ng’a ZW, Lang’at KB, Kirui MC, Gonoi T, Bii CC. The Presence of Mycotoxins in Kenya’s Kalenjin Traditional Fermented Milk “Mursik”. J Sci Res Rep. 2018;21(1):1–8. doi: 10.9734/jsrr/2018/7500

[40] Ghasemi-Kebria F, Joshaghani H, Taheri NS, Semnani S, Aarabi M, Salamat F, et al. Aflatoxin contamination of wheat flour and the risk of esophageal cancer in a high risk area in Iran. Cancer Epidemiol. 2013;37(3):290–3. doi: 10.1016/j.canep.2013.01.010 23434312

[41] Mureithi W, Den Biggelaar C, Wesakania EW, Kamau K, Gatundu C. Management of trees used in Mursik (fermented milk) production in Trans-Nzoia District, Kenya. J Ethnobiol. 2000.

[42] Hakami R, Mohtadinia J, Etemadi A, Kamangar F, Nemati M, Pourshams A, et al. Dietary intake of benzo(a)pyrene and risk of esophageal cancer in north of Iran. Nutr Cancer. 2008;60(2):216–21. doi: 10.1080/01635580701684831 18444153

[43] Oncina-Cánovas A, Torres-Collado L, García-de-la-Hera M, Compañ-Gabucio LM, González-Palacios S, Signes-Pastor AJ, et al. Association between dairy products consumption and esophageal, stomach, and pancreatic cancers in the PANESOES multi case-control study. Cancers (Basel). 2024;16(24):4151. doi: 10.3390/cancers16244151 39766051 PMC11674531

[44] Elena Doudoladova K, David O, Douglas N. Comparative genetics of alcoholism in the Kenyan populations. Adv J Microbiol Res. 2004. http://internationalscholarsjournals.org/journal/ajmr

